# The Amount of Cross-Linker Influences Affinity and Selectivity of NanoMIPs Prepared by Solid-Phase Polymerization Synthesis

**DOI:** 10.3390/polym16040532

**Published:** 2024-02-16

**Authors:** Valentina Testa, Laura Anfossi, Simone Cavalera, Fabio Di Nardo, Thea Serra, Claudio Baggiani

**Affiliations:** Laboratory of Bioanalytical Chemistry, Department of Chemistry, University of Torino, Via Giuria 7, 10125 Torino, Italy; v.testa@unito.it (V.T.); laura.anfossi@unito.it (L.A.); simone.cavalera@unito.it (S.C.); fabio.dinardo@unito.it (F.D.N.); thea.serra@unito.it (T.S.)

**Keywords:** molecular imprinting, nanoMIP, rabbit IgG, cross-linking, N,N′-methylene-bis-acrylamide, binding isotherm, binding affinity, binding selectivity

## Abstract

The cross-linker methylene-bis-acrylamide is usually present in nanoMIPs obtained by solid-phase polymerization synthesis at 2 mol% concentration, with very few exceptions. Here, we studied the influence of variable amounts of methylene-bis-acrylamide in the range between 0 (no cross-linker) and 50 mol% concentration on the binding properties of rabbit IgG nanoMIPs. The binding parameters were determined by equilibrium binding experiments and the results show that the degree of cross-linking defines three distinct types of nanoMIPs: (i) those with a low degree of cross-linking, including nanoMIPs without cross-linker (0–05 mol%), showing a low binding affinity, high density of binding sites, and low selectivity; (ii) nanoMIPs with a medium degree of cross-linking (1–18 mol%), showing higher binding affinity, low density of binding sites, and high selectivity; (iii) nanoMIPs with a high degree of cross-linking (32–50 mol%), characterized by non-specific nanopolymer–ligand interactions, with low binding affinity, high density of binding sites, and no selectivity. In conclusion, the results are particularly relevant in the synthesis of high-affinity, high-selectivity nanoMIPs as they demonstrate that a significant gain in affinity and selectivity could be achieved with pre-polymerization mixtures containing quantities of cross-linker up to 10–20 mol%, well higher than those normally used in this technique.

## 1. Introduction

The interest of the scientific community in molecularly imprinted polymers (MIPs) has progressively moved from micrometer-sized materials to particles of much smaller dimensions. Imprinted nanoparticles, or “nanoMIPs”, show many advantages, including good solubility in buffers, reduced non-specific binding, and faster mass transfer and binding kinetics due to a larger surface/mass ratio [1,2]. NanoMIPs can be prepared by controlled living radical polymerization [3], distillation [4], mini- or micro-emulsion [5,6], or precipitation [7], but all these approaches show significant drawbacks, including complex or cumbersome purification methods to isolate nanoparticles from the polymerization mixture and fully eliminate the template molecule.

Solid-phase polymerization synthesis (SPPS) represents an innovative approach to the preparation of molecularly imprinted nanoparticles, or “nanoMIPs” [8]. In this approach, polymerization happens in the interstitial space between loosely packed glass beads grafted with the template molecule. The formation of cross-linked polymeric chains at the interface with the glass surface results in the imprinting of nanoparticles by the grafted template molecules [9]. At the end of the polymerization process, the non-covalent interaction between nanoMIPs and grafted template molecules is strong enough to allow any residual monomers, polymerization by-products, and low-affinity polymers to be easily removed by washing the glass beads with a weak solvent—typically cold water—whereas high-affinity nanoMIPs are subsequently eluted by washing with a stronger solvent able to break the non-covalent molecular interactions. When performed in water, the SPPS approach has shown its validity for very different types of polar templates, such as small organic molecules [10,11,12,13,14], peptides and proteins [15,16,17,18,19], polysaccharides [20,21,22], nucleic acids [23], viruses [24,25], and whole cells [26]. 

Despite the great variety of target molecules, the SPPS technique uses a pre-polymerization mixture whose composition seems to be rather fixed and evidently inspired by the seminal work of Hoshino et al. [7]. Typically, it is composed of acrylic acid (AA) and/or N-(3-aminopropyl)methacrylamide (AMPA) as functional monomers capable of establishing ionic and hydrogen bonding interactions with the template, t-butylacrylamide (tBAM) and isopropylacrylamide (NIPAM) as moderately hydrophobic and thermoresponsive co-monomers, respectively [8]. Some reported exceptions involve the use of different functional monomers such as acrylamide [20,27,28,29], N,N-diethylaminoethylmetacrylate [27,30], 2-hydroxyethylmetacrylate [31], 2-(methacryloxy)ethyl)trimethylammonium chloride [20,28], (4-acrylamidophenyl)aminomethaniminium acetate [21], or ethylene glycol methacrylate phosphate [11,32,33]. Concerning the cross-linker, BIS at 2 mol% concentration is greatly prevalent, and very few papers report the use of alternative cross-linkers, as ethylene-bis-acrylamide [15,21,34,35] or N,O-methacryloylethanolamine, ethylene glycol dimethacrylate, and glycerol dimethacrylate [35]. Interestingly, there are some reports of nanoMIPs prepared in the presence of higher molar concentrations of BIS, at 16% molar concentration [20,27,30], or completely without a cross-linker, in the so-called linear molecularly imprinted polymers (LMIPs) approach [28,36].

Intrigued by the observation that until now the effect of different amounts of cross-linker has never been reported in detail, we decided to study the effect of variable amounts of BIS—in the range between 0 (no cross-linker) and 50 mol% concentration—in pre-polymerization mixtures on the binding properties of rabbit IgG (rIgG) nanoparticles prepared as previously reported [35]. 

## 2. Materials and Methods

### 2.1. Chemicals and Materials

Acrylic acid (AA), ammonium persulphate (APS), bovine IgG (bIgG), (EDAC), N-hydroxysuccinimide (NHS), N-isopropylacrylamide (NIPAm), N,N′-methylene-bis-acrylamide (BIS), rabbit IgG (rIgG), N-tertbutylacrylamide (TBAm), N,N,N′,N′-tetramethylethylendiamine (TEMED), and trifluoroacetic acid (TFA) were purchased from Sigma-Merck (Milan, Italy). Solvents and all other chemicals were purchased from Sigma-Merck (Milan, Italy). Ultrapure water was obtained with a Purelab Prima System from Elga (Marlow, UK). Stock solutions of rIgG and bIgG were prepared by dissolution of 25 mg of protein in 25 mL of phosphate buffer (20 mmol L^−1^, 0.13 mol L^−1^ NaCl, pH 7.4) and stored in the dark at −20 °C. Coomassie Blue G250 protein assay reagent was from VWR International (Milan, Italy).

### 2.2. Synthesis of NanoMIPs 

Pre-polymerization mixtures (overall monomer concentration: 1.3 mmol L^−1^) were prepared by mixing increasing amounts of cross-linker and conveniently decreasing amounts of functional monomers (fixed molar ratio AA:NIPAM:TBAm = 10:15:24) in 25 mL of ultrapure water, as reported in Table 1. 

Then, 5 mL of mixture was introduced into 50 mL polypropylene SPE cartridges filled with 2.5 g of glass beads (Spheriglass-2429, 70–100 μm average particle size, Potters, UK) covalently grafted with rIgG as previously reported [35]. The cartridges were deoxygenated by sparging with nitrogen for 5 min; then, 3 μL of TEMED and 100 μL of 30 mg mL^−1^ aqueous solution of APS were added to the sealed cartridges with a syringe and polymerization was carried out at room temperature for 60 min in a roller-equipped incubator. The supernatant was eliminated by vacuum aspiration, cartridges were cooled to 4 °C, and low-affinity nanoMIPs and polymerization by-products were washed with 10 × 2 mL of ice-cold water. High-affinity nanoMIPs were recovered by eluting the cartridges at room temperature with 5 × 2 mL of 0.1 mol L^−1^ aqueous TFA. The eluates were dried in rotavapor, weighted, and stored at room temperature.

A non-imprinted linear polymer without cross-linker was obtained in the same experimental conditions in terms of the composition of the polymerization mixture and polymerization time, but without the presence of functionalized glass beads. After the polymerization, the solution was filtered on a 0.22 μm nylon membrane, dried, weighted, and stored at room temperature. The attempt to prepare non-imprinted polymers in the presence of increasing amounts of BIS failed because of their tendency to form opalescent suspensions that filtered on 0.22 μm nylon membranes invariably resulted in substantial loss of polymers. 

NanoMIPs were covalently grafted onto aminated glass beads in accordance with the protocol previously reported with minor modifications [12]. In 4 mL glass vials, 1 mg of nanoMIPs was dissolved under sonication in 1 mL of N,N-dimethylformamide. Then, 5 mg of NHS (0.043 mmol), and 7 mg of DIC (0.047 mmol) were added and the solution was incubated at 4 °C for 2 h. Then, it was transferred into a 10 mL flask containing 1 g of aminated glass beads in 4 mL of phosphate buffer (0.1 mol L^−1^, pH 7.4). The suspension was incubated at room temperature overnight, filtered on 0.22 μm nylon membrane, washed with ultrapure water, rinsed twice with acetone, dried under vacuum at room temperature, and stored in the dark at 4 °C.

### 2.3. Measurement of Size and Charge of NanoMIPs

The size and zeta potential of hydrodynamic particles were measured with a ZetaViews Nanoparticle Tracking Analyzer PMX-120, (Analytik, Cambridge, UK) using a laser source at 488 nm. Solid samples of each of the nanoMIPs were dissolved in working dilution with phosphate buffer (20 mmol L^−1^, 0.13 mol L^−1^ NaCl, pH 7.4) under sonication and about 2 mL of the sample was immediately injected into the analyzer. The results are the average of three distinct measurements made at 25 ± 0.1 °C.

### 2.4. Measurement of NanoMIPs Binding Properties 

Binding isotherms were measured onto 40 mg of exactly weighed glass beads supporting nanoMIPs in 4 mL flat-bottom amber glass vials. Then, 1.0 mL of phosphate buffer containing increasing amounts of rIgG or bIgG ranging from 1 to 50 µg mL^−1^ was added. Vials were incubated under continuous agitation on a horizontal rocking table overnight at room temperature. After that, the solutions were filtered on 0.22 μm nylon membranes and the free amounts of proteins were measured by Bradford assay in accordance with the previously reported protocol [35]. Each experimental point was assessed as the average of three repeated measures.

Binding parameters were calculated by using TableCurve 2D 5.0 (Systat Software Inc., San Josè, CA, USA). Non-linear least squares fitting was applied to the averaged experimental data. Binding isotherm parameters were calculated by using a Langmuir binding isotherm model:

(1) $$B=\frac{B_{max}K_{eq}F}{1+K_{eq}F}$$

where B is the protein bound to the polymer (nmol g^−1^), F is the protein not bound to the nanoMIPs (µmol L^−1^), B_max_ the binding site density (nmol g^−1^), and K_eq_ the apparent equilibrium binding constant (mol L^−1^). To assure robust results, weighted (1/y) Pearson VII limit minimization was used as the minimization method. To avoid incorrect results due to fit trapped in local minima, the numerical process was carried out several times by using different initial guess values for the binding parameters.

## 3. Results and Discussion

After drying, nanoMIPs were collected as white solids, with yields calculated with respect to the amount of monomers in polymerization mixtures of 1.1–2.0% (1–1.9 mg). When dissolved in phosphate buffer under sonication, nanoMIPs gave completely transparent solutions, without any perceivable turbidity. Because of the limited quantity of nanoparticles obtained, no attempts were made to establish the effective degree of cross-linking. Therefore, as a first approximation, it was assumed that it does not differ significantly from the amount of BIS introduced in the pre-polymerization mixtures.

### 3.1. Size and Charge of NanoMIPs

The hydrodynamic diameter, d_p_ (Table 2), was measured by laser nanoparticle tracking in phosphate buffer at pH 7.4. It shows nanoparticles with average diameters around 100 nm, ranging from 90 nm (P50) to 168 nm (P05), and with a polydispersity index between 0.26 (P50) and 0.38 (P0), which corresponds to moderately polydispersed nanoparticles. It must be noted that for nanoMIPs with a very low degree of cross-linking (P0–P1), the estimated hydrodynamic diameter appears to be essentially independent from the amount of BIS introduced in the pre-polymerization mixture, while for more cross-linked nanoMIPs (P2–P8), it decreases decisively and then stabilizes for polymers with a higher degree of cross-linking (P32–P50).

Concerning nanoparticle charge, acrylic acid is the only charged functional monomer which gives nanoMIPs the properties of charged (anionic) polyelectrolytes at pH 7.4. This is supported by ζ potential measurements (Table 2), where, at pH 7.4, all the nanoMIPs show a net negative potential, with ζ values between −10.5 mV (P50) and −24.9 mV (P0). Also in this case, for nanoMIPs with a very low degree of cross-linking (P0–P2), the ζ potential appears to be essentially independent from the degree of cross-linking, but it progressively decreases in parallel with the decrease in the amount of acrylic acid present in the polymers with the highest degree of cross-linking (P4–P50). 

### 3.2. Binding Affinity of NanoMIPs

**Figure 1:**
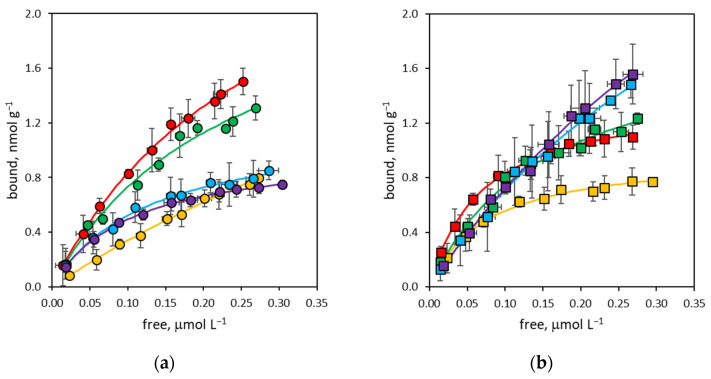
Binding isotherm curves for rabbit immunoglobulin G (rIgG). (**a**) NanoMIPs containing from 0% to 2% BIS (yellow: P0; red: P02; green: P05; blue: P1; violet: P2); (**b**) nanoMIPs containing from 4% to 50% BIS (yellow: P4; red: P8; green: P18; blue: P32; violet: P50). Error bars: ±1 s.d.

**Figure 2:**
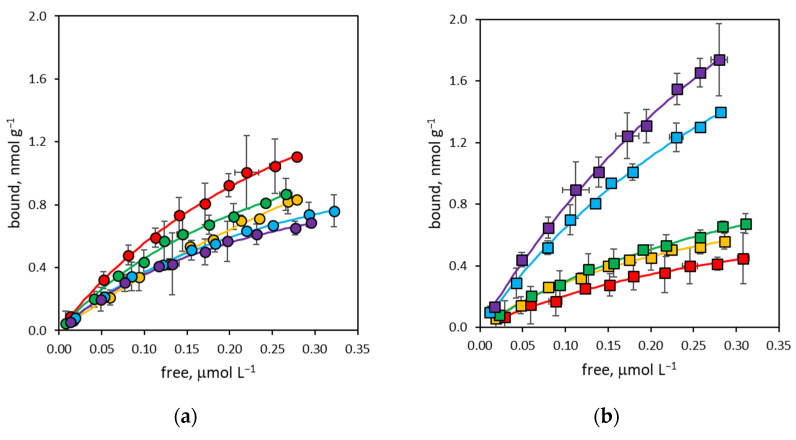
Binding isotherm curves for bovine immunoglobulin G (bIgG). (**a**) NanoMIPs containing from 0% to 2% BIS (yellow: P0; red: P02; green: P05; blue: P1; violet: P2); (**b**) nanoMIPs containing from 4% to 50% BIS (yellow: P4; red: P8; green: P18; blue: P32; violet: P50). Error bars: ±1 s.d.

In traditional molecular imprinting techniques, the cross-linker constitutes up to 90 mol% of the polymerization mixture, thus exerting a leading effect not only on the morphology of the polymer, but also on its binding properties [37,38]. On the contrary, in the SPPS technique, the cross-linker is usually present in a very limited amount, practically never exceeding 2 mol%, and the main contribution to the molecular recognition properties is expected to be coming from the functional monomers that predominate in the pre-polymerization mixtures. However, observing the free-to-bound curves for rIgG (Figure 1) and bIgG (Figure 2), although presenting the characteristic shape of Langmuirian binding isotherms, they do not show an easily interpretable trend. Consequently, it is evident that the degree of cross-linking has a direct effect on the binding properties of the corresponding nanoMIPs, influencing both binding site density and the apparent equilibrium binding constant of nanoparticles. 

**Figure 3:**
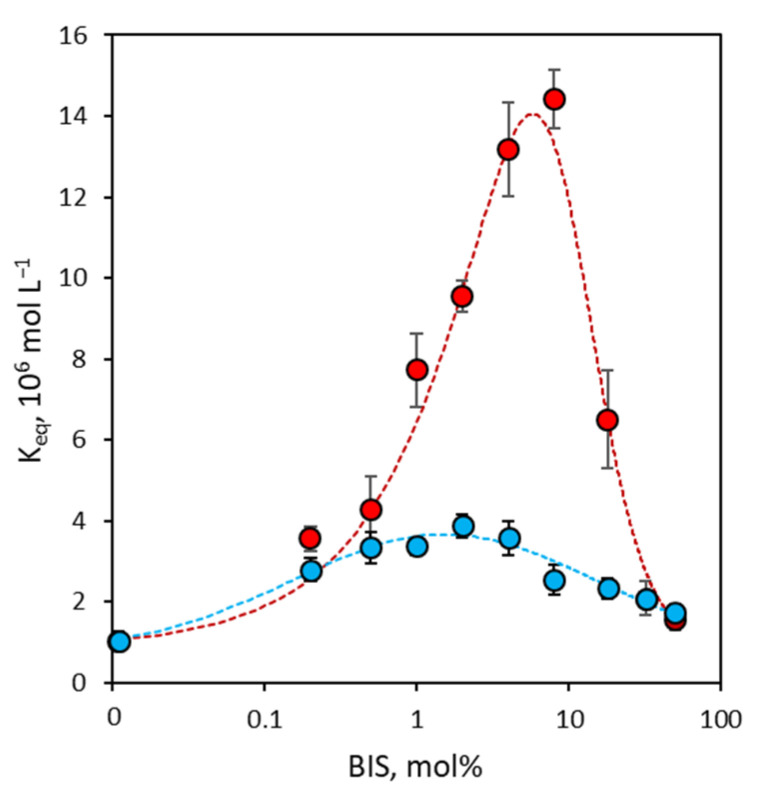
Apparent equilibrium binding constants (K_eq_) measured for rIgG (red circles) and bIgG (blue circles). Error bars: ±1 s.d.

The determination of the apparent equilibrium binding constants through the fitting of the binding curves with a simple Langmuirian binding model allows for a clearer evaluation of the dependence of nanoMIP binding properties on the degree of cross-linking. In Figure 3, it is possible to observe that even the linear polymer (P0) prepared without BIS shows affinity towards the rIgG template, with an apparent equilibrium binding constant of 1.0 ± 0.2 × 10^6^ mol L^−1^, lower than that of all other nanoMIPs considered in this study but higher than the value for rIgG measured in the equivalent non-imprinted linear polymer prepared in the absence of a template (K_eq_~10^5^ mol L^−1^, estimated as the upper limit for bound-to-free partition experiment, see Figure S1). It must be observed that this result seems to confirm the feasibility of imprinted linear polymers through the SPPS approach previously reported in literature [28,36].

For cross-linked nanoMIPs (P02–P50), binding affinity shows a well-defined trend: it begins to increase in parallel with the increase in the degree of cross-linking, going from 3.6 ± 0.3 × 10^6^ mol L^−1^ for P02 up to 14.4 ± 0.7 × 10^6^ mol L^−1^ for P8, thus spanning almost an order of magnitude. Then, it gradually decreases for nanoMIPs with a higher degree of cross-linking (P18–P50) until reaching a low value of 1.6 ± 0.3 × 10^6^ mol L^−1^, which for the P50 nanoMIP is comparable with that of the non-cross-linked nanoMIP. 

It can be hypothesized that this trend depends on the rigidity of the polymer structures due to the different degrees of cross-linking. Since it is assumed that in nanoMIPs, the binding between ligands and nanoparticles is strengthened by the rearrangement of the polymer structure in the binding sites around the ligand [9,39], for nanoMIPs with a low degree of cross-linking, this affinity is low since, due to excessive chain flexibility, this rearrangement is not stable enough to contribute to the increase in binding affinity. As the degree of cross-linking increases, the three-dimensional structure of the polymer becomes proportionally more rigid, thus increasing the structural stability of the binding sites around the ligands. Beyond a certain degree of cross-linking, which can tentatively be identified at around 10 mol%, the polymer structure becomes so rigid as to make it difficult to rearrange the binding sites around the ligands, thus causing a decrease in binding affinity.

The binding of bIgG presents a similar trend to that of rIgG. Also in this case, the linear polymer (P0) prepared without BIS shows a low apparent equilibrium binding constant of 1.0 ± 0.2 × 10^6^ mol L^−1^; then, affinity increases slightly up to 3.9 ± 0.3 × 10^6^ mol L^−1^ for the P2 nanoMIP and progressively decreases until it coincides with the low affinity observed for rIgG for polymers with a higher degree of cross-linking (P32–P50). As the quantitative differences between rIgG and bIgG’s binding affinity for nanoMIPs define the binding selectivity of the polymers, it can therefore be anticipated that this depends on the degree of cross-linking (vide infra). 

### 3.3. Binding Site Density of NanoMIPs

**Figure 4:**
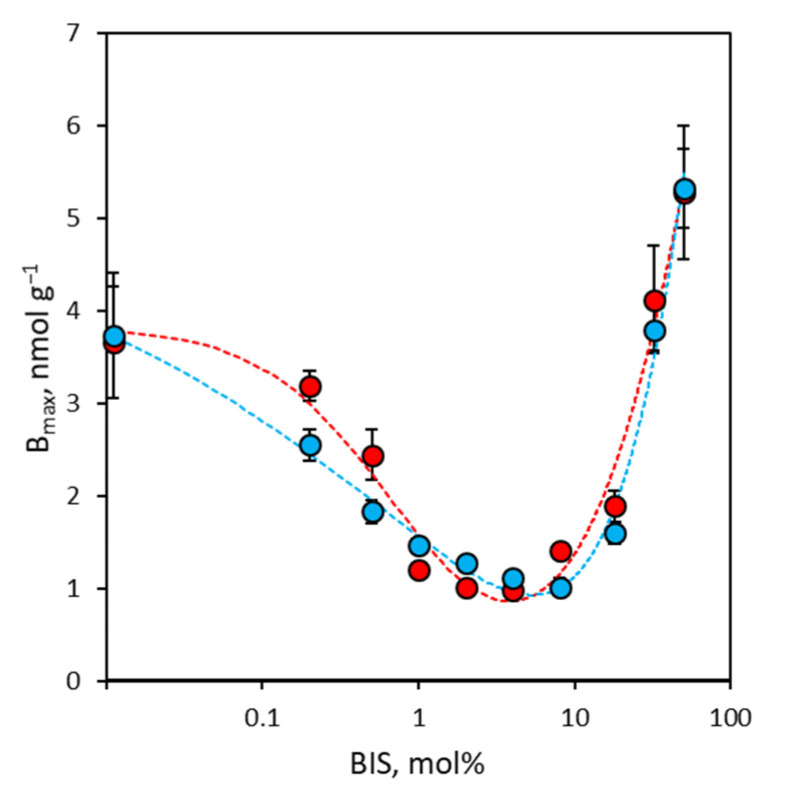
Binding site density (B_max_) measured for rIgG (red circles) and bIgG (blue circles). Error bars: ±1 s.d.

Concerning binding site density, the results of laser nanoparticle tracking show that there is an inverse relationship between the degree of cross-linking and the average size of the nanoMIPs. Therefore, since it is plausible to retain that the number of binding sites per single nanoparticle depends on the mean size of the nanoMIPs, and considering that covalent grafting for all nanoMIPs was performed starting from identical concentrations of the different nanoparticles, it is reasonable to assume that the coverage of the glass in terms of binding site density is the same for all nanoMIPs and, thus, the measured binding site densities truly correspond to the density of binding sites accessible on the surface of the glass spheres after covalent grafting of the nanoMIPs. In Figure 4, it is possible to observe that both rIgG and bIgG share the same trend, where binding site density decreases as the degree of cross-linking increases, going from 3.7 nmol g^−1^ (P0) to a minimum value of 1.0 nmol g^−1^ (P4, measured for rIgG, P8, measured for bIgG), and then it increases steadily up to 5.3 nmol g^−1^. The decrease in binding site density as binding affinity increases has been well known in molecular imprinting for a long time [40,41], but more puzzling is the increase in binding site density accompanied by a complete loss of selectivity (vide infra) in nanoMIPs with a higher degree of cross-linking (P18–P50). It can be hypothesized that the progressive loss of high-affinity binding sites controlling the features of the binding isotherm makes the residual weak non-specific interactions between IgG molecules and the surface of the nanoMIPs predominant and experimentally observable [42]. 

### 3.4. Binding Selectivity of NanoMIPs

Considering an imprinted polymer, for two ligands that have a measurable binding affinity for the same binding sites, at the equilibrium, binding selectivity α can be measured by comparing the binding isotherms in conditions far from saturation (i.e., for low values of free ligands). In these conditions, Equation (1) simplifies to:

(2) $$B=B_{max}K_{eq}F$$

where the product B_max_ × K_eq_ is the binding capacity β of the imprinted polymer for a given ligand (note that in this work, the products of binding capacity for the concentration of free ligand measured at the lowest point of the binding isotherms ranged from 0.03 to 0.3 for rIgG and from 0.02 to 0.07 for bIgG). Thus, considering the template rIgG and the ligand bIgG, the binding selectivity α of nanoMIPs can be defined as:

(3) $$\alpha =\frac{\beta_{bIgG}}{\beta_{rIgG}}$$

where β_bIgG_ and β_rIgG_ are the binding capacities calculated for bIgG and rIgG, respectively.

**Figure 5:**
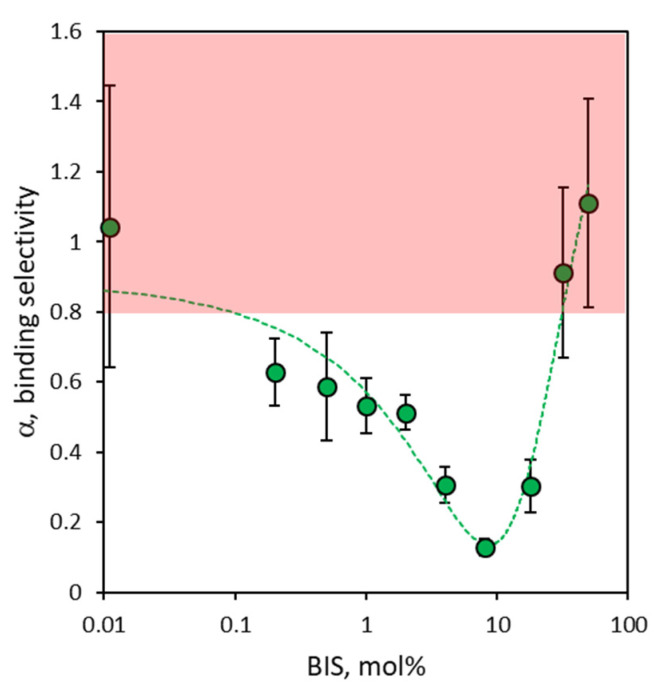
Green circles: binding selectivity (α) measured as the ratio between binding capacities (β) of nanoMIPs for bIgG and rIgG. Error bars: ±1 s.d. The pale red area indicates no (α ≥ 1) or marginal (0.8 ≤ α < 1) binding selectivity.

As seen in Section 3.3, both rIgG and bIgG show very similar values of binding site concentrations across the entire composition range of nanoMIPs considered; thus, binding selectivity can essentially be attributed to the contribution given by different values of apparent equilibrium binding constants for the pair of ligands. Of consequence, the magnitude of binding affinity for the template rIgG is the key factor controlling binding selectivity. In Figure 5, it is possible to observe that non-cross-linked (P0) and highly cross-linked (P32–P50) nanoMIPs—characterized by low values of binding affinity for rIgG—show no (α ≥ 1) or marginal (0.8 ≤ α < 1) binding selectivity. For the same reason, nanoMIPs with intermediate degrees of cross-linking (P02–P18) show a clear relationship between binding affinity for rIgG—and thus the degree of cross-linking—and binding selectivity, as the latter begins to decrease in parallel with the increase in the degree of cross-linking, going from 0.63 ± 0.10 for P02 up to a minimum of 0.13 ± 0.02 for P8. Then, it gradually increases again to reach an absence of binding selectivity for highly cross-linked nanoMIPs.

## 4. Conclusions

The experimental results reported here show a remarkable effect of the degree of cross-linking on the bonding properties of nanoMIPs. In fact, while—as easily predictable—dimensions and charge decrease (d_p_ from 165 nm to 90 nm and ζ from −24.9 mV to −10.5 mV, respectively) as the degree of cross-linking increases, binding properties change in a more complex way than expected, with a trend that may presumably be due to how the stiffness of the nanoMIPs influences the behavior of the imprinted binding sites. The effect of the degree of cross-linking on binding properties can therefore be summarized by identifying three distinct types of nanoMIPs depending on the amount of cross-linking agent added in the pre-polymerization mixture: (i) nanoMIPs with a low degree of cross-linking, including nanoMIPs prepared in the absence of a cross-linking agent (P0–P05), characterized by a low binding affinity (K_eq_ = 1.0 ± 0.2 × 10^6^ mol L^−1^—4.3 ± 0.8 × 10^6^ mol L^−1^), high density of binding sites (B_max_ = 2.5 ± 0.3 nmol g^−1^—3.7 ± 0.6 nmol g^−1^), and low selectivity (α = 0.59–1.04); (ii) nanoMIPs with a medium degree of crosslinking (P1-P18), characterized by higher binding affinity (K_eq_ = 6.5 ± 1.2 × 10^6^ mol L^−1^—14.4 ± 0.7 × 10^6^ mol L^−1^), low density of binding sites (B_max_ = 1.2 ± 0.1 nmol g^−1^—1.9 ± 0.2 nmol g^−1^), and high selectivity (α = 0.30–0.53); (iii) nanoMIPs with a high degree of cross-linking (P32-P50), characterized by low binding affinity (K_eq_ = 1.6 ± 0.3 × 10^6^ mol L^−1^—2.1 ± 0.4 × 10^6^ mol L^−1^), high density of binding sites (B_max_ = 4.1 ± 0.6 nmol g^−1^—5.3 ± 0.7 nmol g^−1^)—presumably due to the predominance of non-selective interactions between nanopolymers and ligands—and no selectivity (α = 0.91–1.11).

In conclusion, we think that these experimental results are particularly relevant from the point of view of the synthesis of high-affinity, high-selectivity nanoMIPs because they demonstrate that a significant gain in affinity and selectivity could be achieved simply through the use of pre-polymerization mixtures containing quantities of cross-linking agent up to 10–20 mol%, i.e., well higher than those normally used in the SPPS approach.

## Figures and Tables

**Table 1:** Composition of pre-polymerization mixtures calculated for a 25 mL solution.

NanoMIP	BIS, mol%	BIS, µmoles	AA, µmoles	NIPAM, µmoles	TBAm, µmoles
P0	0	-	66.3	99.5	159.1
P02	0.2	0.65	66.2	99.3	158.9
P05	0.5	1.63	66	99	158.4
P1	1	3.25	65.7	98.59	157.6
P2	2	6.5	65	97.5	156
P4	4	13	63.7	95.5	152.8
P8	8	26	61	91.5	146.5
P18	18	58.5	54.4	81.6	130.5
P32	32	104	45.1	67.7	108.2
P50	50	162.5	33.2	49.7	79.6

**Table 2:** Hydrodynamic diameter (d_p_) ± 1 s.d., polydispersity index (PDI), and zeta potential (ζ) measured for nanoMIPs.

NanoMIP	BIS, mol%	d_p_ (nm)	PDI	ζ (mV)
P0	0	165 ± 68	0.38	−24.9
P02	0.2	163 ± 66	0.32	−23.7
P05	0.5	168 ± 67	0.30	−24.4
P1	1	160 ±77	0.31	−23.5
P2	2	141 ± 65	0.28	−24.5
P4	4	129 ± 53	0.29	−21.6
P8	8	109 ± 40	0.28	−17.4
P18	18	96 ± 22	0.27	−14.5
P32	32	90 ± 20	0.29	−13.4
P50	50	90 ± 17	0.26	−10.5

## Data Availability

The raw and processed data required to reproduce these findings are available on request.

## Supplementary Materials

The following supporting information can be downloaded at: https://www.mdpi.com/article/10.3390/polym16040532/s1, Figure S1: Binding isotherm of rIgG to non-imprinted linear polymer (BIS = 0%).
