# Supplementary material for: The Amount of Cross-Linker Influences Affinity and Selectivity of NanoMIPs Prepared by Solid-Phase Polymerization Synthesis
Source: Polymers (Basel). 2024 Feb 16;16(4):532. doi: 10.3390/polym16040532 (PMC10892272; doi:10.3390/polym16040532)
Supplement: Supplementary file 1 [file polymers-16-00532-s001.zip › polymers-2826905-supplementary.pdf]

Supplementary Materials

Figure S1: binding isotherm of rIgG to non-imprinted linear polymer (BIS = 0%)

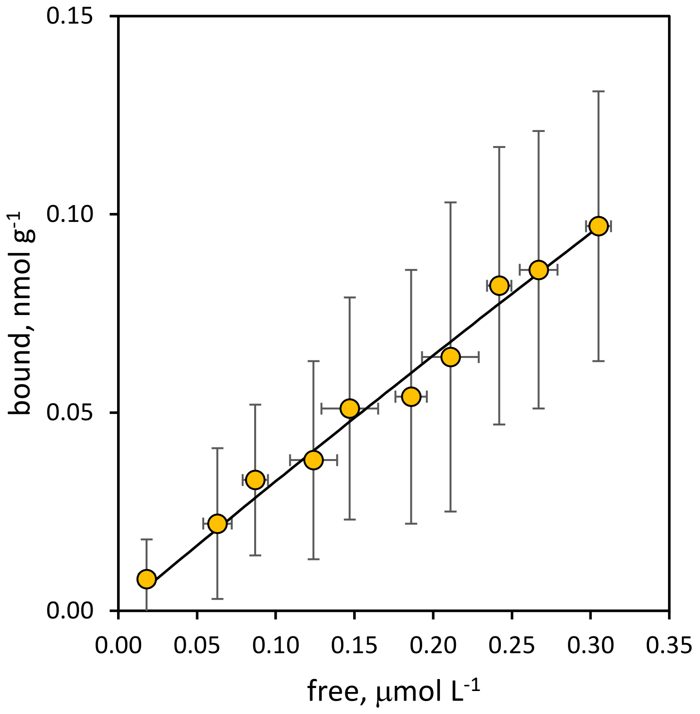

n = 10 (mean of 3 replicates)

correlation coefficent (adjusted for degree of freedom): $r^2 = 0.981$

fit standard error: 0.00379

fit F-value: 520.4

B_{max}: 2.27 ± 5.20 nmol g^{-1} (t = 0.436; P > t 0.674)

K_{eq}: 0.15 ± 0.35 x 10^6 L mol^{-1} (t = 0.422; P > t 0.684)
